# Optimization of Pre‐Treatments to Overcome Stickiness‐Related Hurdles and Enhance Drying Efficiency in Apple Pomace

**DOI:** 10.1002/fsn3.70988

**Published:** 2025-10-29

**Authors:** Hyo Jun Won, Ae‐Jin Choi

**Affiliations:** ^1^ Food Tech Resources Research Division, National Institute of Crop and Food Science (NICS), Rural Development Administration (RDA) Wanju‐Gun Republic of Korea

**Keywords:** apple pomace, drying efficiency, enzyme, ethanol, pre‐treatments, stickiness

## Abstract

Apple pomace (AP), a major by‐product of apple juice processing, poses environmental and economic challenges because of its high moisture content, leading to storage difficulties and microbial degradation. Moreover, the high sugar content of AP contributes to stickiness during drying, which causes material agglomeration and limits efficient moisture removal—representing a significant hurdle in the drying process. Sustainable utilization of AP requires efficient drying methods to enhance its stability and usability. This study hypothesizes that enzymatic and ethanol pre‐treatments can significantly improve the drying efficiency of AP by alleviating stickiness‐related barriers. The objective was to optimize pre‐treatment conditions to maximize moisture removal while potentially maintaining the quality of dried AP. Experiments were conducted using different ethanol concentrations, enzyme types (Pectinase, Cellulase), and treatment durations, with drying efficiency evaluated based on the removed moisture content (RMC) under various pre‐treatment conditions. Under optimal conditions—combining Pectinase and Cellulase enzyme treatments with ethanol pre‐treatment—the RMC increased from 12% (control) to 67%, representing a 5.5‐fold improvement in drying efficiency. These findings demonstrate that optimized pre‐treatments can effectively mitigate stickiness‐induced drying hurdles, enhance AP drying efficiency, reduce waste, and support sustainable food processing. The proposed approach contributes to minimizing the environmental footprint of the apple processing industry. Future research should focus on scaling up these pre‐treatment techniques and assessing their economic feasibility for industrial applications.

## Graphical abstract

This study presents an approach to mitigate stickiness and improve drying efficiency in apple pomace valorization. Pre‐treatment strategies were optimized to enhance moisture removal, while acknowledging the importance of physicochemical quality for future study. The proposed workflow supports sustainable utilization of apple pomace as a functional food material.

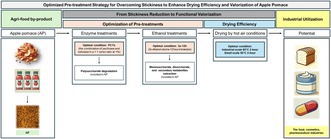

## 1 Introduction

The food processing industry generates substantial quantities of waste, presenting significant environmental challenges due to the accumulation and disposal of by‐products such as peelings, seeds, and pomace (Fierascu et al. 2020; Taghian Dinani and van der Goot 2023; Zaky et al. 2024). The need to valorize these by‐products is paramount, as their effective utilization not only mitigates environmental impacts but also enhances the nutritional profile of consumer products (Anand and Barua 2022; Dawood et al. 2022; Ofei Darko et al. 2024). Transforming by‐products into valuable products not only reduces waste but also contributes to the circular economy by repurposing waste into valuable resources (Scarano et al. 2022). This aligns with global sustainability goals, including the United Nations' Sustainable Development Goals (SDGs), particularly SDG 12 (Responsible Consumption and Production) and SDG 13 (Climate Action) (Lal 2022; Yudhistira et al. 2023; AfsahHejri et al. 2025). Applying sustainable practices in food processing can provide significant environmental benefits, including a reduced carbon footprint from waste disposal and improved resource efficiency. In particular, reducing waste decreases greenhouse gas emissions from landfills and alleviates the burden on waste management systems (De Boni et al. 2022; Marimuthu et al. 2024). Despite the potential benefits, agri‐food by‐products are frequently undervalued, which accounts for approximately 14% of global food loss during post‐harvest stages (Cassani and Gomez‐Zavaglia 2022; Taghian Dinani and van der Goot 2023; Pandey et al. 2025). Optimizing the valorization of these by‐products is crucial for sustainable food systems (Costa et al. 2022; Hobbi et al. 2023; LigardaSamanez et al. 2025).

Apple pomace (AP), the solid residue remaining after the extraction of juice from apples, represents a significant by‐product of the apple processing industry (Asif et al. 2024; Venkidasamy et al. 2024). This by‐product is abundant in bioactive compounds, making it a valuable resource for various applications, including animal feed, biofuel production, and as a raw material for food products (Hobbi et al. 2023; Kandemir et al. 2022; Kauser et al. 2024; Zhang et al. 2021). However, the high moisture content of AP poses significant challenges for its storage and utilization, leading to rapid microbial degradation and loss of valuable nutrients (Asif et al. 2024; Hasan et al. 2024; Singh and Singh 2022). Reducing the moisture content of AP is essential to inhibit microbial activity and preserve nutrients (Aslam et al. 2024; Araujo et al. 2024; Tulej and Głowacki 2022). Efficient utilization of AP can minimize waste and enhance resource efficiency, thereby reducing its environmental footprint. To address these challenges and improve the storage stability and usability of AP, sustainable and efficient drying technologies are essential (Jangam 2011).

Hot air drying, a widely used method, operates at relatively low temperatures such as 50°C and 60°C and is known for its energy efficiency and ability to preserve the quality of the dried product (Acar et al. 2020; Calin‐Sanchez et al. 2020; Indiarto et al. 2021; Yin et al. 2025). This method is particularly advantageous because it maintains the nutritional and sensory qualities of the dried product, which are crucial for both consumer acceptance and nutritional integrity. Additionally, hot air drying is an effective solution for large‐scale operations and industrial applications, offering low operating costs and the ability to reduce environmental impact (Chua and Chou 2003; Deng et al. 2019; Hu, Dutta, and Srilatha 2021; Kim et al. 2024). Studies have shown that the energy consumption of hot air drying is significantly lower compared to other drying methods such as freeze drying (Acar et al. 2020; Al Faruq et al. 2025; Mu et al. 2025). Furthermore, hot air drying is commonly conducted at temperatures around 50°C in industrial settings, which balances energy efficiency and product quality retention (Li et al. 2024; Paraman et al. 2015; Senadeera et al. 2020). This highlights the potential for hot air drying as a sustainable and cost‐effective method for drying agri‐food by‐products, including AP.

However, hot air drying also presents certain limitations, particularly in the drying of apple pomace. Because of its high moisture and fiber content, AP tends to form a dense structure during drying, leading to reduced airflow penetration and non‐uniform moisture removal. This can result in prolonged drying times and increased energy consumption, potentially offsetting its cost‐effectiveness (Araujo et al. 2024; Beigi 2015; Deng et al. 2019).

Additionally, the high sugar content in AP contributes to stickiness during drying, which can lead to material agglomeration and impede efficient moisture evaporation (Fernandes et al. 2021; Pujapanda et al. 2025; Won et al. 2024). This stickiness can also contribute to uneven drying and product adhesion to drying surfaces, further complicating process efficiency. Furthermore, excessive exposure to heat may lead to the degradation of heat‐sensitive bioactive compounds, such as polyphenols and vitamins, which are abundant in AP (Acar et al. 2020; Fernandes et al. 2021; Araujo et al. 2024). Extended drying at elevated temperatures can also result in undesirable changes in texture, color, and flavor, negatively affecting the final product quality (Calin‐Sanchez et al. 2020; Llavata et al. 2024).

These limitations necessitate the exploration of effective pre‐treatment techniques to enhance the drying performance of AP. To address these challenges, pre‐treatment methods such as enzymatic hydrolysis and ethanol treatment have been studied to improve drying efficiency and mitigate quality degradation during hot air drying (Costa et al. 2022; Deng et al. 2019; Du et al. 2021; Kim et al. 2024).

Recent advancements indicate that pre‐treatment methods, such as enzymatic and ethanol processes, significantly improve the drying efficiency of various agri‐food by‐products (Bassey et al. 2021; Iranshahi et al. 2023; Macedo et al. 2025; Yuan et al. 2025). These methods modify the physical and chemical properties of the material, improving moisture removal rates and reducing drying time (Alberici et al. 2020; Awasthi et al. 2021; Macedo et al. 2025). For instance, enzymatic treatments break down complex carbohydrates, facilitating more efficient moisture removal (Cano‐Lamadrid and Artes‐Hernandez 2021; Martau et al. 2021; Patel et al. 2022; Toy et al. 2022), while ethanol processes alter the microstructure of AP, improving its drying characteristics (Awasthi et al. 2021; Fernandes et al. 2021; Llavata et al. 2024). Moreover, previous studies have reported that similar enzyme‐ and ethanol‐based pre‐treatments can improve drying kinetics and may help preserve nutritional and bioactive compounds in the final dried product, thereby supporting the quality and functionality of dried AP (Costa et al. 2022; Kim et al. 2024; Tepe 2024). The integration of pre‐treatment techniques can lead to significant energy savings and cost reductions in the drying process, enhancing its economic viability and environmental sustainability (Golebiewska et al. 2022).

This study aims to optimize the pre‐treatment conditions to enhance the drying efficiency of AP, thereby improving the sustainability of the apple processing industry. By investigating the effects of various ethanol concentrations, enzyme types, and treatment durations on the drying rate of AP, we seek to identify the optimal conditions that can significantly improve drying efficiency. Optimizing these drying conditions can reduce the environmental impact associated with AP disposal and promote its potential utilization across various industries that use agri‐food by‐products as raw materials.

## 2 Materials and Methods

### 2.1 Apple Pomace

The apple pomace (AP) used in this study was provided by Chungcheongbuk‐do Horticulture Nonghyup (Chungcheongbuk‐do, South Korea). The pomace was composed of the Fuji apple variety, which is predominantly used for juice production in South Korea. The AP included peel, seeds, and residual pulp remaining after juice extraction. The juice was extracted using a mechanical pressing method, which is widely applied in industrial juice processing.

To maintain freshness and prevent microbial growth, the collected AP was transported to the laboratory under frozen conditions (below −20°C). Upon arrival, the AP was immediately stored in a freezer maintained at below −20°C to ensure its quality until further processing.

### 2.2 Reagents

The enzymes Pectinase (Pectinex), Cellulase (Celluclast), and Viscozyme (Viscozyme L), which aid in the breakdown of complex carbohydrates, were purchased from Novozymes, and ethanol was purchased from Ethanol Supplies World Co. Ltd. These reagents were selected to enhance the drying efficiency of AP and were used in various concentrations and combinations during the pre‐treatment process.

### 2.3 Quality Changes During Storage

#### 2.3.1 Moisture Content

The moisture content of AP thawed at refrigerated temperature and room temperature was measured by drying 100 g of the sampled material at 105°C for 4 h. *W*_1_ represents the weight of the sample before drying (g), and *W*_2_ represents the weight of the sample after drying (g). The moisture content (%, *w*/*w*) was calculated using the following equation:

$$\text{Moisture content}\;\left(\%\right)=\left(W_{1}-W_{2}\right)/W_{1}\times 100$$

#### 2.3.2 pH Measurement

To measure the pH, 100 g of AP was mixed with 100 mL of distilled water and homogenized using a blender. The mixture was then centrifuged at 4000 rpm for 20 min at 4°C. After centrifugation, 50 mL of the supernatant was collected and the pH was measured using a pH meter (Orion Star A211, Thermo Scientific).

### 2.4 Pre‐Treatment Conditions

The pre‐treatment conditions for optimizing the drying efficiency of AP were based on the method by Kim et al. (2024). The optimal enzyme treatment conditions (1% Cellulase and Viscozyme at a 1:1 *w*/*w* ratio) and ethanol treatment conditions (4 times volume, 4°C, 24 h) were either combined or modified. The experimental conditions for optimizing the pre‐treatment to enhance the drying efficiency of AP are shown in Table 1. Experiments were conducted with AP fully thawed in the refrigerator for 2 days, ensuring no drip formation after thawing. Drying efficiency was evaluated based on the removed moisture content.

**TABLE 1:** Treatment conditions for enhancing the drying efficiency of AP.

Group	Treatments	Enzyme treatment			Ethanol treatment
		Enzyme 1% (CV^1^)	50°C (humid chamber)	25°C (room temperature)	
Control^2^		—	—	—	—
(A)^4^	T1^3^	O	O	—	—
	T2	—	O	—	—
	T3	O	—	O	—
	T4	—	—	O	—
(B)	T5	O	O	—	O
	T6	—	O	—	O
	T7	O	—	O	O
	T8	—	—	O	O
	T9	—	—	—	O

^1^ CV: 1:1 (*w*/*w*) ratio of Cellulase and Viscozyme.

^2^ Control: non‐treatment.

^3^ T1: 100 g thawed sample was treated with enzyme 1% and the enzyme reaction was conducted in a humid chamber set at 50°C for 2 h with 95% relative humidity, and ethanol was added 4 times and left for 24 h at room temperature. T6: The reaction of a 100 g thawed sample was performed in a humid chamber set at 50°C for 2 h with 95% relative humidity, and ethanol was added 4 times and left for 24 h at room temperature. T7: 100 g thawed sample was treated with enzyme 1% and the enzyme reaction was carried out at room temperature, 25°C, covered with foil for 24 h, and ethanol was added 4 times and left for 24 h at room temperature. T8: 100 g thawed sample was conducted at room temperature, 25°C, covered with foil for 24 h, T5: 100 g thawed sample was treated with enzyme 1% and the enzyme reaction was conducted in a humid chamber set at 50°C for 2 h with 95% relative humidity and ethanol was added 4 times was added and left for 24 h at room temperature, T6: The reaction of a 100 g thawed sample was performed in a humid chamber set at 50°C for 2 h with 95% relative humidity and ethanol 4 times was added and left for 24 h at room temperature, T7: 100 g thawed sample was treated with enzyme 1% and the enzyme reaction was carried out at room temperature, 25°C, covered with foil for 24 h and ethanol 4 times was added and left for 24 h at room temperature, T8: 100 g thawed sample was conducted at room temperature, 25°C, covered with foil for 24 h and ethanol 4 times was added and left for 24 h at room temperature, T9: Ethanol was added four times to 100 g of the thawed sample and left at room temperature for 24 h.

^4^ (A) and (B) are groups classified with or without ethanol treatment. Treatment procedures were performed in three replications (*n* = 3).

The experimental conditions were designed as follows: T1–T9 groups were subjected to various combinations of enzyme and ethanol treatments. For instance, in T1, 100 g of thawed AP was treated with 1% enzyme and incubated in a humid chamber at 50°C with 95% relative humidity for 2 h, while in T5, the same conditions were applied, but ethanol was added, and the sample was kept at room temperature for 24 h. In the T7 group, the enzyme treatment was carried out at room temperature (25°C) for 24 h, followed by ethanol treatment for an additional 24 h. The groups were classified based on the presence or absence of ethanol treatment and were designated as groups (A) and (B). All experiments were conducted in triplicate.

#### 2.4.1 Ethanol Concentration

To determine the optimal ethanol treatment conditions, 100 g of thawed AP was treated with ethanol at 1, 2, 3, and 4 times the weight of the sample at room temperature (25°C) for 1, 4, 8, 12, and 24 h. After treatment, the samples were vacuum filtered (Whatman 4, 180 mm), and the drying efficiency was evaluated based on the removed moisture content of the remaining AP.

#### 2.4.2 Enzyme Type and Time

To determine the optimal enzyme treatment conditions, experiments were designed based on pre‐treatment process optimization (T5, T7) and optimal ethanol treatment conditions (3×, 12 h). The study involved a series of experiments using different enzyme types and treatment durations, with the enzyme concentration fixed at 1% (*w*/*w*), which was selected based on preliminary trials indicating optimal hydrolysis efficiency and drying performance. The pre‐treatment optimization considered enzyme specificity, hydrolysis efficiency, and its impact on moisture removal.

Each treatment used 100 g of AP, treated with 1% (*w*/*w*) of Pectinase (P), Cellulase (C), Viscozyme (V), or their combinations: Pectinase and Cellulase (PC = 1:1), Pectinase and Viscozyme (PV = 1:1), Cellulase and Viscozyme (CV = 1:1), and Pectinase, Cellulase, and Viscozyme (PCV = 1:1:1). The effect of enzyme treatment duration was assessed by incubating AP samples for 0, 1, 2, 4, 8, 12, 16, and 24 h under predefined conditions. The optimal enzyme treatment duration was determined based on the removed moisture content (RMC), considering the point at which moisture removal reached a plateau while ensuring industrial feasibility for large‐scale applications.

For incubation conditions, T5 treatments were performed in a humid chamber at 50°C with 95% relative humidity for 2 h, while T7 treatments were conducted at room temperature (25°C) for 24 h. After enzyme treatment, samples were subjected to ethanol pre‐treatment (3× sample weight) at 25°C for 12 h, followed by vacuum filtration. The removed moisture content (RMC) was measured at different time points to evaluate drying efficiency. Table 1 summarizes the detailed experimental conditions.

#### 2.4.3 Drying Condition

To determine the optimal drying temperature, the T7_PC_2 treatment group, identified as optimal in experiments, was used. Drying was conducted using a hot air drying oven with a constant airflow rate. The samples were evenly distributed on trays to ensure uniform drying. Drying was conducted at 50°C, 60°C, 70°C, and 80°C, with continuous air circulation to facilitate moisture removal. The drying efficiency was evaluated based on the removed moisture content and drying rate at each temperature.

### 2.5 Evaluation of Drying Efficiency

#### 2.5.1 Removed Moisture Content (RMC)

The removed moisture content (RMC) was calculated to assess the drying efficiency of the samples. This value represents the percentage of moisture removed from the samples during the drying process. Samples were measured using a dryer (ThermoStable PF‐305, Daihan Scientific) at 50°C, and the weight of the samples was recorded at 1‐, 2‐, 4‐, 6‐, and 24‐h intervals to determine the amount of moisture removed. RMC was calculated using the formula:

$$\mathrm{RMC}\;\left(\%\right)=\left(\left[W_{1}-W_{2}\right]/W_{1}\right)\times 100$$

where *W*_1_ is the weight of the sample before drying, and *W*_2_ is the weight of the sample after drying. This equation quantifies the percentage of moisture removed relative to the initial sample weight.

#### 2.5.2 Drying Rate

The drying rate was calculated at 50°C based on the weight of the samples before and during drying. The equation used is as follows:

$$\text{Drying rate}\;\left(g\;\text{water}/g\;\mathrm{dry}\;\text{base}\right)=\left(Mt+dt-Mt\right)/dt$$

where M*t* + d*t* represents the moisture content of the sample at *t* + d*t*, and M*t* represents the moisture content of the sample at drying time *t*.

### 2.6 Statistical Analysis

All experiments were conducted in triplicate, and statistical analysis was performed using R Studio (version 4.4.3). Data were expressed as mean values ± SD. Differences between groups were analyzed using one‐way ANOVA, followed by Duncan's multiple range test for post hoc comparisons at a significance level of *p* < 0.05. For comparisons between two groups, a t‐test was used with significance levels set at *p < 0.05, **p < 0.01, and ***p < 0.001.

## 3 Results

### 3.1 Moisture Content and pH

**FIGURE 1:**
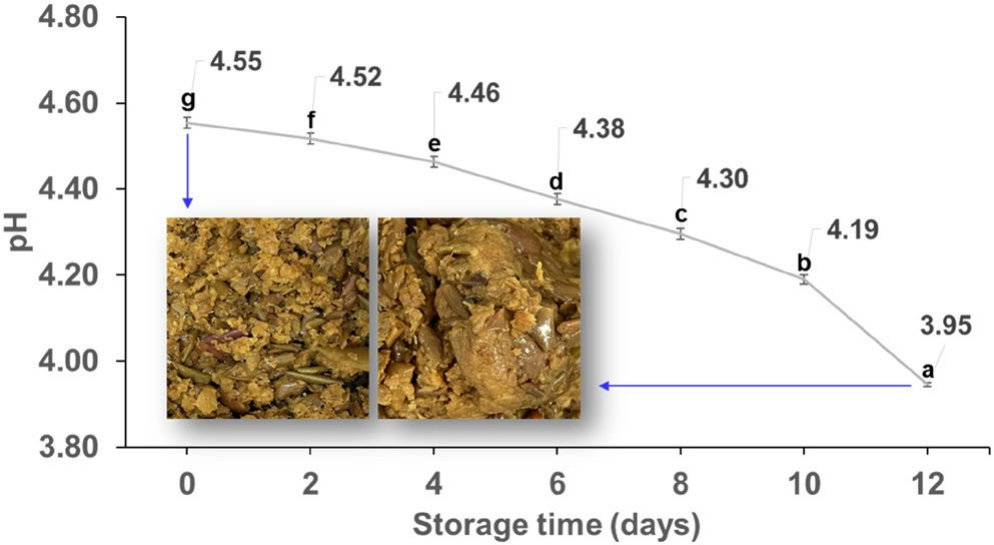
pH changes in AP over storage time. Different small letters above each value indicate significant differences in drying times determined using Duncan's multiple‐range test. Statistical analyses were performed by R Studio (version 4.3.3). Values are the mean ± SD of three replications (*n* = 3).

The results for moisture content and appearance of frozen AP thawed at refrigerated and room temperatures are shown in Table 2. The moisture content of the pomace at both conditions was found to be 77%–78%. The pH results over the storage period are depicted in Figure 1. The pH of the AP was 4.55 ± 0.01 on day 0, gradually decreasing over time. Remarkably, between days 10 and 12, the pH dropped significantly, reaching its lowest value of 3.95 ± 0.01 on day 12, indicating a more than 10% decrease from the initial value.

**TABLE 2:** Moisture content by thawing temperature.

Refrigerated temperature (4°C)
78.40 ± 0.10
Appearance^1^ 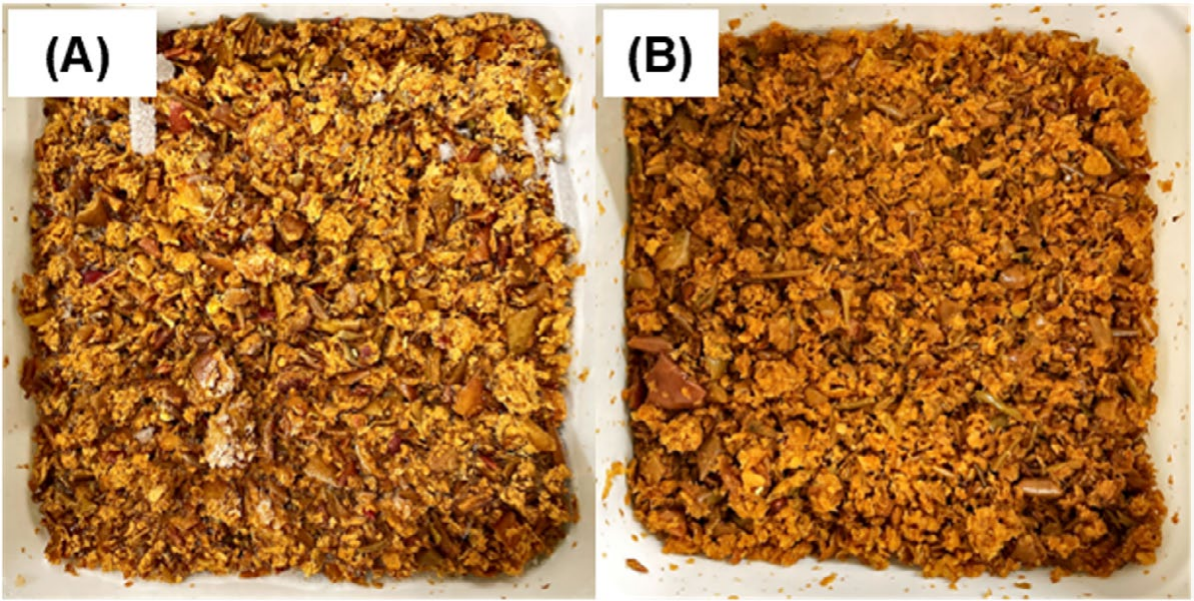

^1^ (A): before drying, (B): after drying.

### 3.2 Pre‐Treatment

**FIGURE 2:**
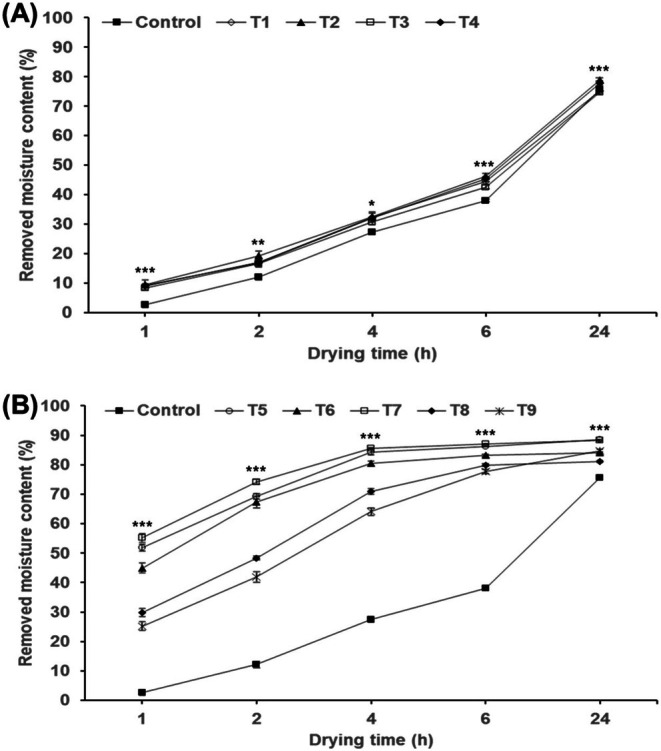
Comparison of RMC depending on treatment methods. Details of the treatment methods for (A) and (B) are provided in the group section of Table 1. Different * above each mark indicate significant differences in drying time by Duncan's multiple range test. Each treatment group (including control) according to drying time was all ***. Statistical analyses were performed by R Studio (version 4.3.3). Values are the mean ± SD of three replications (*n* = 3). *p < 0.05, **p < 0.01, ***p < 0.001.

The results for drying efficiency based on pre‐treatment conditions of AP are shown in Figure 2. Treatments including ethanol (B) demonstrated significantly higher drying efficiency compared to enzyme treatments (A). After 2 h of hot air drying at 50°C, the T5 treatment (enzyme treatment at 50°C for 2 h followed by ethanol treatment) showed a moisture removal of 69%, which is 5.8 times higher than the control (12%). The T7 treatment (enzyme treatment at 25°C for 2 h followed by ethanol treatment) showed a 74% moisture removal, 6.2 times higher than the control. Consequently, T5 and T7 were selected for further detailed condition optimization, with T7 considered more suitable for large‐scale industrial processes due to its room temperature enzyme treatment.

### 3.3 Ethanol Treatment Time

**FIGURE 3:**
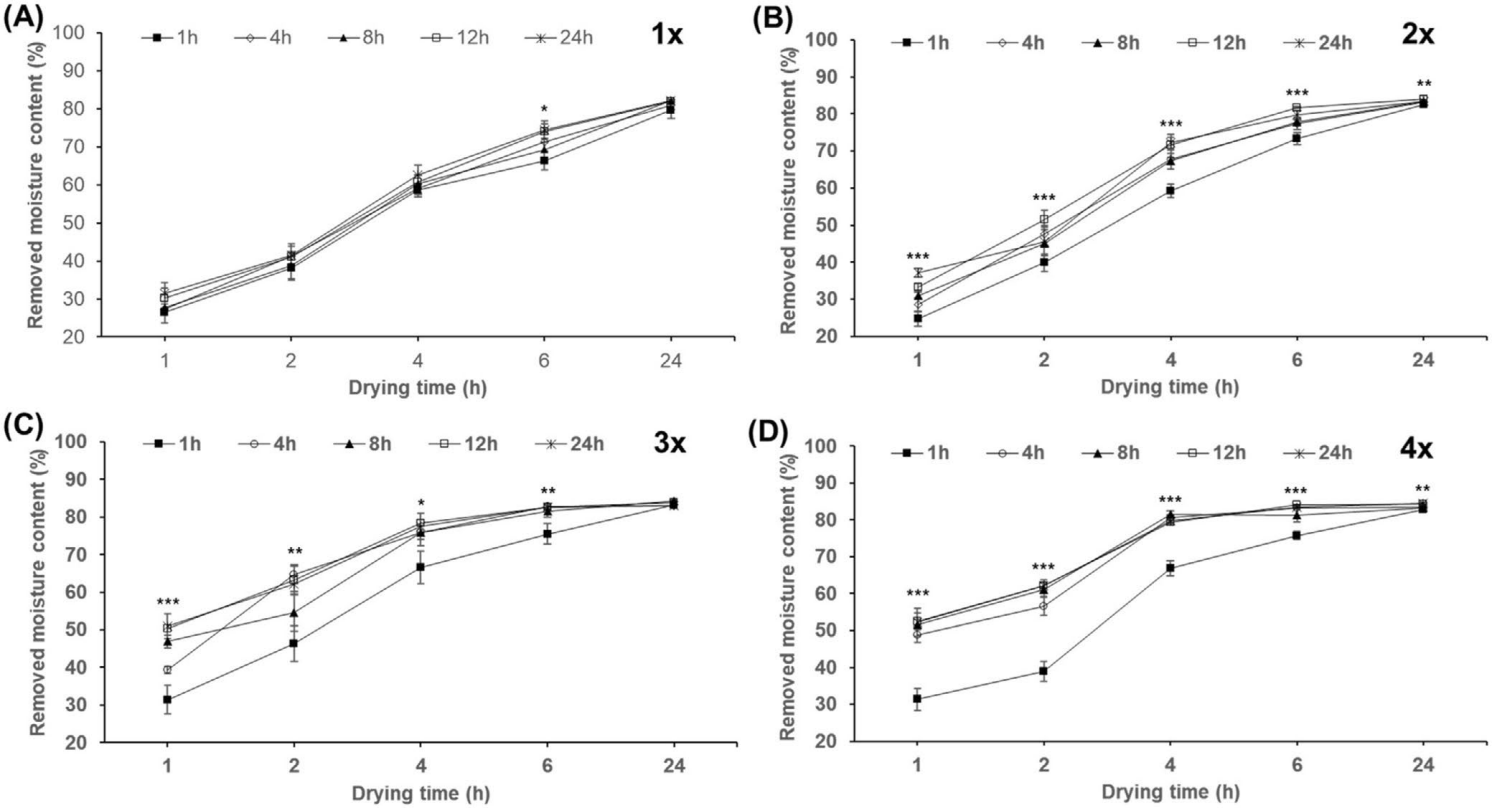
Comparison of RMC depending on ethanol treatment ratios. (A) is a graph comparing ethanol “1 multiples (×1, compared to 100 g of sample)” treatment at each immersion time. (B), (C), and (D) are graphs comparing “2 multiples (×2, compared to 100 g of sample),” “3 multiples (×3, compared to 100 g of sample),” and “4 multiples (×4, compared to 100 g of sample)” treatments, respectively. Different * above each mark indicate significant differences in drying time by Duncan's multiple range test. Each treatment group (including control) according to drying time was all ***. Statistical analyses were performed by R Studio (version 4.3.3). Values are the mean ± SD of three replications (*n* = 3). *p < 0.05, **p < 0.01, ***p < 0.001.

The results for drying efficiency based on ethanol treatment time and volume are shown in Figure 3. As the ethanol treatment time increased and the ethanol volume ratio increased from 1× to 4×, the drying efficiency improved. However, for more than the 3 × 12‐h treatment condition, no significant additional improvements in drying efficiency were observed. After 2 h of hot air drying at 50°C, the 3 × 12‐h treatment showed a moisture removal of 63%, which was 1.4 × higher than the 3 × 1‐h treatment (46%) and 1.5 times higher than the 1 × 12‐h treatment (41%). Therefore, the optimal ethanol treatment condition was determined to be 3 × for 12 h, considering both efficiency and economic factors. Subsequent experiments were conducted using these conditions.

### 3.4 Enzyme Time

**FIGURE 4:**
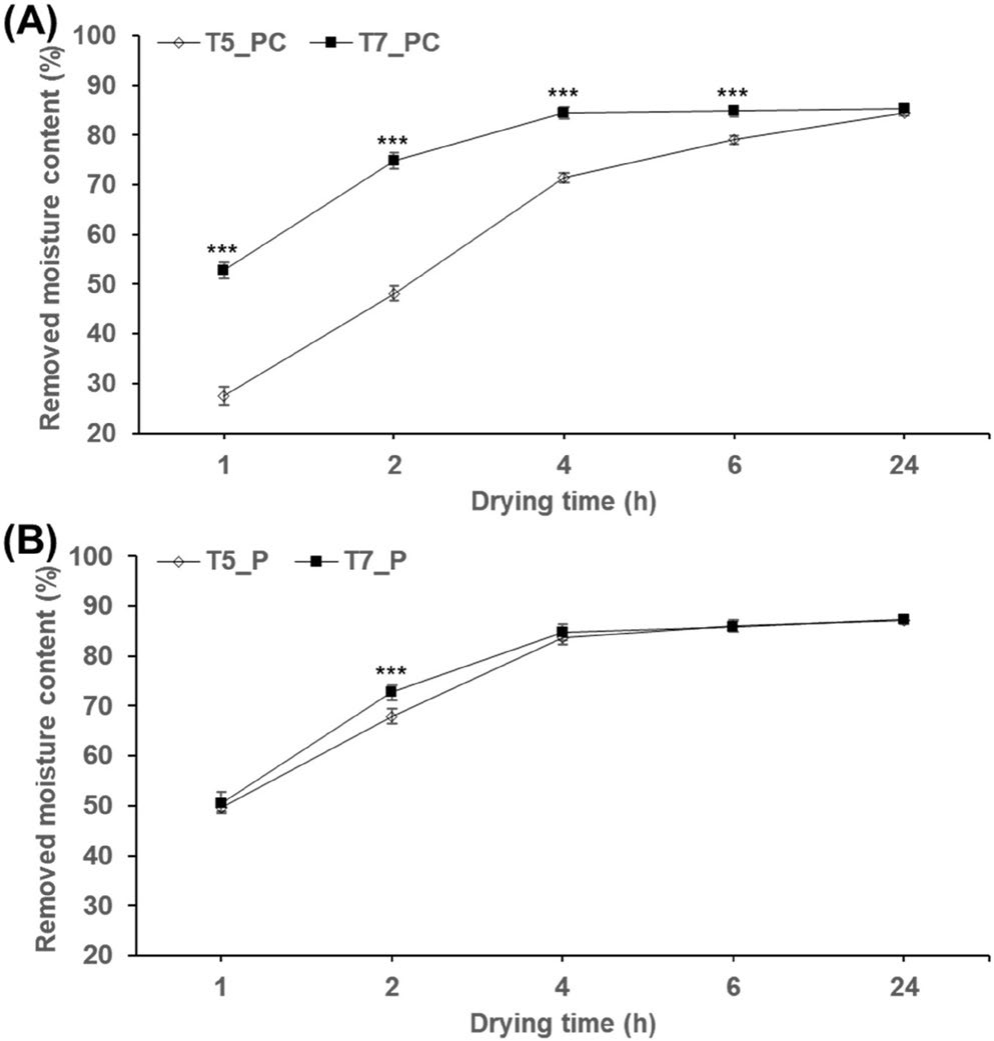
Comparison of RMC for T5 and T7 treated with P and PC. T5 and T7 with P and PC treatments are the same as in Table 3. Different * above each mark indicate a significant difference in drying time determined using the *t*‐test. Each treatment group (including control) according to drying time was all *** (determined using Duncan's multiple‐range test). Statistical analyses were performed by R Studio (version 4.3.3). Values are the mean ± SD of three replications (*n* = 3). *p < 0.05, **p < 0.01, ***p < 0.001.

The results for drying efficiency based on the type of enzyme treatment are shown in Table 3 and Figure 4. In the T5 treatment group, Pectinase (1%) showed the highest drying efficiency, while in the T7 treatment group, both Pectinase (P) and the combination of Pectinase and Cellulase (PC, 1:1 *w*/*w* ratio, 1%) showed high efficiency. In all enzyme treatment groups, T7 had significantly higher drying efficiency than T5. After 2 h of hot air drying at 50°C, the T7_PC treatment showed a moisture removal of 75%, which was 1.6 times higher than the T5_PC treatment (48%). Considering the dietary fiber composition of AP, including pectin, cellulose, and hemicellulose, PC was selected as the optimal enzyme. Therefore, the T7 method and PC enzyme were selected as the treatment conditions (T7_PC) for subsequent experiments.

**TABLE 3:** Comparison of RMC depending on enzyme types for T5 and T7 treatments.

Treatments	Drying time (h)	Enzyme types							*F*‐value
		P^2^	C	V	PC	CV	PV	PCV	
		RMC^3^							
T5^1^	1	^c^49.72^4^ ± 0.86^A^ ^5^	^e^21.98 ± 0.86^E^	^e^27.94 ± 1.33^D^	^e^27.54 ± 1.77^D^	^d^27.30 ± 2.17^D^	^e^33.11 ± 2.14^C^	^e^38.86 ± 1.86E^E^	63.625***
	2	^b^67.93 ± 1.21^A^	^d^47.88 ± 1.44^C^	^d^54.03 ± 1.37^B^	^d^48.17 ± 1.45^C^	^c^49.53 ± 1.31^C^	^d^53.06 ± 1.71^B^	^d^54.87 ± 1.67^B^	44.561***
	4	^a^83.65 ± 0.61^A^	^c^70.32 ± 1.43^D^	^c^76.50 ± 1.07^B^	^c^71.47 ± 0.98^CD^	^b^73.99 ± 1.27^bc^	^c^75.16 ± 1.03^B^	^c^74.87 ± 1.15^B^	30.587***
	6	^a^86.11 ± 0.98^A^	^b^78.53 ± 1.23^A^	^b^83.35 ± 0.95^C^	^b^79.04 ± 0.93^C^	^a^83.14 ± 1.19^B^	^b^81.63 ± 0.86^B^	^b^83.11 ± 0.45^B^	14.863***
	24	^a^87.10 ± 0.53^AB^	^a^84.69 ± 0.72^CD^	^a^86.69 ± 0.57^A^	^a^84.55 ± 0.58^CD^	^a^84.01 ± 0.77^D^	^a^85.07 ± 0.51^CD^	^a^85.79 ± 0.26^bc^	7.346**
	*F*‐value	412.005***, ^6^	960.675***	891.667***	768.819***	603.587***	507.497***	513.123***	
T7	1	^d^50.58 ± 1.48^A^	^e^26.76 ± 2.17^D^	^e^31.61 ± 1.06^C^	^c^52.76 ± 1.56^A^	^c^52.92 ± 1.31^A^	^d^29.99 ± 1.52^CD^	^d^36.25 ± 1.87^B^	104.092***
	2	^c^72.70 ± 0.76^AB^	^d^42.51 ± 1.51^F^	^d^46.55 ± 1.02^E^	^b^74.85 ± 1.65^A^	^b^71.85 ± 1.35^B^	^c^52.37 ± 0.99^D^	^c^64.89 ± 0.74^C^	254.047***
	4	^b^84.72 ± 0.69^A^	^c^67.29 ± 1.58^C^	^c^66.55 ± 1.58^C^	^a^84.45 ± 1.20^A^	^a^84.10 ± 1.09^A^	^b^77.75 ± 0.79^B^	^b^83.30 ± 0.59^A^	102.528***
	6	^ab^85.90 ± 0.47^A^	^b^78.07 ± 1.08^C^	^b^81.47 ± 0.61^B^	^a^84.82 ± 0.99^A^	^a^85.49 ± 0.94^A^	^a^85.25 ± 0.54^A^	^ab^85.05 ± 0.31^A^	29.158***
	24	^a^87.41 ± 0.52^A^	^a^83.49 ± 0.38^C^	^a^86.46 ± 0.49^A^	^a^85.32 ± 0.64^B^	^a^86.20 ± 0.55^AB^	^a^86.47 ± 0.47^A^	^a^86.27 ± 0.17^AB^	13.211***
	*F*‐value	650.183**	543.013***	1027.582***	243.960***	341.323***	1359.054***	1010.009***	

^1^ T5 and T7 treatment methods are the same as those in Table 1.

^2^ P: Pectinase 1%, C: Cellulase 1%, V: Viscozyme 1%, PC: 1:1 Pectinase to Cellulase ratio (1%, *w*/*w*), CV: 1:1 Cellulase to Viscozyme ratio (1%, *w*/*w*), PV: 1:1 Pectinase to Viscozyme ratio (1%, *w*/*w*), PCV: 1:1:1 Pectinase to Cellulase to Viscozyme ratio (1%, *w*/*w*/*w*).

^3^ RMC: removed moisture content (%).

^4^ Different small letters above each value indicate significant differences in drying times determined using Duncan's multiple‐range test.

^5^ Different capital letters above each value indicate significant differences in drying times determined using Duncan's multiple‐range test.

^6^ Statistical analyses were performed by R Studio (version 4.3.3). Values are the mean ± SD of three replications (*n* = 3).

* *p* < 0.05.

** *p* < 0.01.

*** *p* < 0.001.

### 3.5 Enzyme Optimization

**FIGURE 5:**
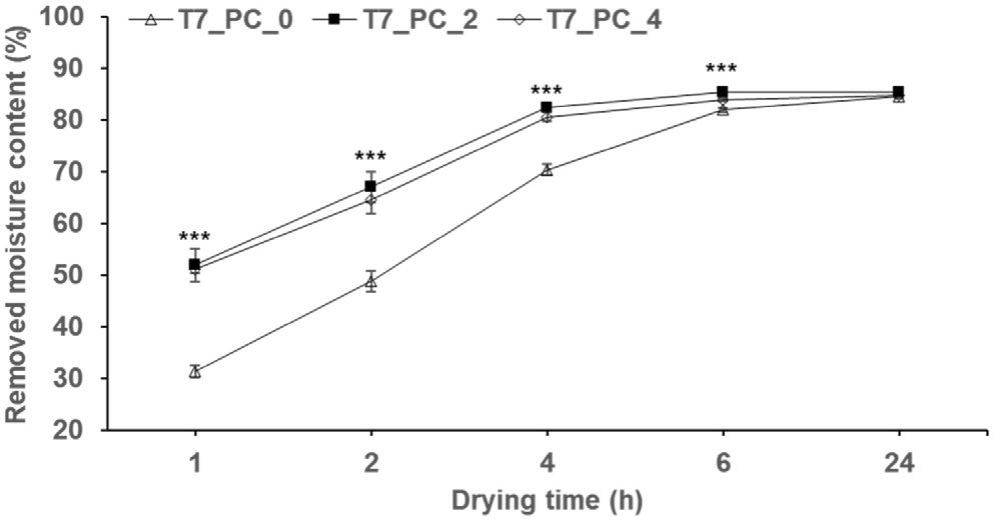
Comparison of RMC at enzyme treatment times 0, 2, and 4 h under T7_PC. T7_PC treatment times (0, 2, and 4 h) are the same as those in Table 4. Different * above each mark indicate a significant difference in drying time determined using the *t*‐test. Each treatment group (including control) according to drying time was all *** (determined using Duncan's multiple‐range test). Statistical analyses were performed by R Studio (version 4.3.3). Values are the mean ± SD of three replications (*n* = 3). *p < 0.05, **p < 0.01, ***p < 0.001.

The results for drying efficiency based on enzyme treatment time are shown in Table 4 and Figure 5. As the enzyme treatment time increased, drying efficiency also increased, but no significant differences were observed for more than 2 h of treatment. Based on these results, the enzyme treatment time was determined to be 2 h. Therefore, the optimal pre‐treatment conditions for enhancing the drying efficiency of AP (T7_PC_2) were determined to be as follows: enzyme treatment with PC at 1% for 2 h at 25°C, followed by ethanol treatment at 3× volume for 12 h at 25°C.

**TABLE 4:** Comparison of RMC by enzyme types for T7‐PC group.

Drying time (h)	Treatment time (h)								*F*‐value
	0	1	2	4	8	12	16	24	
	RMC^1^								
1	^d^31.32^2^ ± 1.11^C^ ^3^	^d^37.90 ± 3.55^B^	^c^51.88 ± 3.22^A^	^d^51.10 ± 0.83^A^	^d^53.63 ± 2.70^A^	^d^52.89 ± 1.69^A^	^c^51.29 ± 1.52^A^	^d^50.28 ± 1.54^A^	27.337***
2	^c^48.70 ± 2.07^B^	^c^52.07 ± 3.46^B^	^b^66.99 ± 3.00^A^	^c^64.55 ± 2.81^A^	^c^63.74 ± 2.40^A^	^c^66.93 ± 2.26^A^	^b^70.01 ± 3.31^A^	^c^68.74 ± 1.85^A^	17.210***
4	^b^70.32 ± 1.12^B^	^b^71.69 ± 3.18^B^	^a^82.30 ± 0.86^A^	^b^80.53 ± 0.88^A^	^b^78.25 ± 1.87^A^	^b^77.98 ± 2.27^A^	^a^80.31 ± 2.49^A^	^b^80.39 ± 0.67^A^	10.896***
6	^a^81.87 ± 0.45^bc^	^a^79.91 ± 2.09^C^	^a^85.24 ± 0.45^A^	^a^83.83 ± 0.32^AB^	^ab^82.27 ± 0.75^B^	^a^82.21 ± 1.23^B^	^a^83.76 ± 0.96^AB^	^ab^82.02 ± 0.40^bc^	5.183**
24	^a^84.45 ± 0.35^B^	^a^84.68 ± 0.54^AB^	^a^85.36 ± 0.43^A^	^a^84.66 ± 0.21^AB^	^a^84.50 ± 0.17^B^	^a^84.55 ± 0.31^AB^	^a^84.72 ± 0.38^AB^	^a^83.42 ± 0.29^C^	4.592**
*F*‐value	730.180***, ^4^	98.603***	105.109***	227.125***	102.992***	116.763***	95.249***	301.842***	

^1^ RMC: removed moisture content (%).

^2^ Different small letters above each value indicate significant differences in drying times determined using Duncan's multiple‐range test.

^3^ Different capital letters above each value indicate significant differences in drying times determined using Duncan's multiple‐range test.

^4^ Statistical analyses were performed by R Studio (version 4.3.3). Values are the mean ± SD of three replications (*n* = 3).

* *p* < 0.05.

** *p* < 0.01.

*** *p* < 0.001.

### 3.6 Selection of Drying Conditions

**FIGURE 6:**
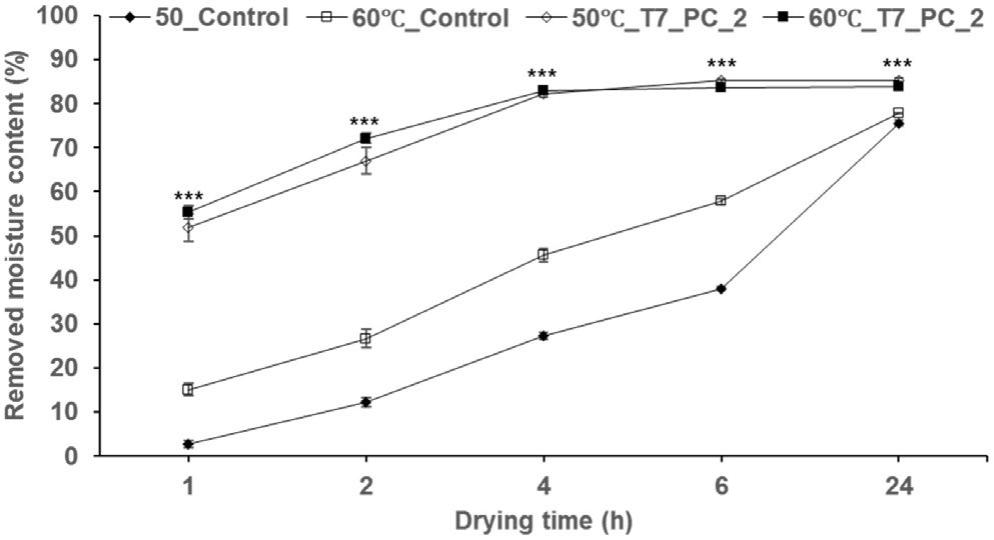
Comparison of RMC between control and T7_PC_2 dried at 50°C and 60°C. Control and T7_PC_2 are the same as in Table 5. Different * above each mark indicate significant differences in drying time determined using the *t*‐test. Each treatment group (including control) according to drying time was all *** (determined using Duncan's multiple‐range test). Statistical analyses were performed by R Studio (version 4.3.3). Values are the mean ± SD of three replications (*n* = 3). *p < 0.05, **p < 0.01, ***p < 0.001.

The results for drying efficiency based on drying temperature for optimally pre‐treated AP are shown in Table 5 and Figure 6. The drying efficiency of the optimal treatment group (T7_PC_2) was significantly higher than that of the control group at all temperatures. After 2 h of drying at 50°C, the RMC of T7_PC_2 was 67%, 5.5 times higher than the control (12.11%). At 60°C for 2 h, it was 2.7 times higher.

**TABLE 5:** Comparison of RMC and drying rate by drying temperature.

Temperature (°C)	Drying time (h)	Control^1^		T7_PC_2^2^		*p*
		RMC^3^	Drying rate	RMC	Drying rate	
50	1	^e^2.66^4^ ± 0.88	0.04	^c^51.88 ± 3.22	0.86	***
	2	^d^12.11 ± 1.03	0.10	^b^66.99 ± 3.00	0.56	***
	4	^c^27.38 ± 0.83	0.11	^a^82.30 ± 0.86	0.34	***
	6	^b^38.05 ± 0.40	0.11	^a^85.24 ± 0.45	0.24	***
	24	^a^75.53 ± 0.70	0.05	^a^85.36 ± 0.43	0.06	***
	*F*‐value	2533.124***, ^5^		105.109		
60	1	^e^15.08 ± 1.45	0.25	^c^55.32 ± 1.57	0.92	***
	2	^d^26.64 ± 2.08	0.22	^b^72.13 ± 1.20	0.60	***
	4	^c^45.66 ± 1.53	0.19	^a^82.84 ± 0.55	0.35	***
	6	^b^57.86 ± 0.71	0.16	^a^83.70 ± 0.27	0.23	***
	24	^a^77.86 ± 0.12	0.05	^a^83.92 ± 0.26	0.06	***
	*F*‐value	668.065***		354.007***		
70	1	^e^19.32 ± 1.49	0.32	^c^59.64 ± 1.86	0.99	***
	2	^d^34.05 ± 2.09	0.28	^b^77.18 ± 1.19	0.64	***
	4	^c^58.22 ± 0.50	0.24	^a^83.40 ± 0.21	0.35	***
	6	^b^70.73 ± 0.98	0.20	^a^83.77 ± 0.35	0.23	***
	24	^a^77.88 ± 0.12	0.05	^a^83.80 ± 0.35	0.06	***
	*F*‐value	784.427***		209.352***		
80	1	^d^32.79 ± 1.88	0.55	^c^60.11 ± 1.53	1.00	***
	2	^c^50.58 ± 0.39	0.42	^b^77.55 ± 1.49	0.65	***
	4	^b^73.85 ± 0.18	0.31	^a^83.85 ± 0.61	0.35	***
	6	^a^78.08 ± 0.16	0.22	^a^84.50 ± 0.20	0.23	***
	24	^a^78.36 ± 0.16	0.07	^a^84.74 ± 0.12	0.06	***
	*F*‐value	924.199***		221.379***		

^1^ Control is the same as those in Table 1.

^2^ T7_PC_2 is the same as those in Table 4.

^3^ RMC: removed moisture content (%).

^4^ Different small letters above each value indicate significant differences in drying times determined using Duncan's multiple‐range test.

^5^ Statistical analyses were performed by R Studio (version 4.3.3). Values are the mean ± SD of three replications (*n* = 3).

* *p* < 0.05.

** *p* < 0.01.

*** *p* < 0.001.

Compared to the control, which required more than 6 h at 50°C and approximately 6 h at 60°C to achieve similar moisture removal, the optimized pre‐treatments reduced the drying time by over 50% at 60°C and more than 60% at 50°C while maintaining drying efficiency. Beyond 4 h, no significant differences were observed at any temperature for T7_PC_2. Therefore, the optimal drying conditions, providing the highest efficiency in the shortest time, were determined to be 60°C for 2 h for industrial‐scale processes and 50°C for 2 h for small‐scale applications.

Considering the moisture content (MC) of AP at 78%, the RMC of 72% after 2 h of drying at 60°C indicates that the sample retained approximately 5% final moisture content.

## 4 Discussion

This study explored the optimal pre‐treatment conditions to maximize the drying efficiency of AP. By applying various ethanol concentrations, enzyme types, and treatment time, we evaluated their impact on drying rates and drew the following key findings.

First, an analysis of moisture content and pH changes demonstrated a continuous decrease in the pH of AP during storage. This phenomenon, linked to increasing acidity, highlights the importance of proper storage conditions to preserve the freshness of AP.

Second, in the optimization of pre‐treatment methods, ethanol treatment proved more effective than enzyme treatment in enhancing drying efficiency. Remarkably, the T7 treatment was found to be suitable for large‐scale industrial processes. This is likely due to the alteration of the physical and chemical structure of AP by ethanol treatment after enzyme treatment, facilitating more efficient moisture removal. Ethanol treatment was found to alter the microstructure of apple pomace, facilitating more efficient moisture removal, which is consistent with the findings of Awasthi et al. (2021) and Costa et al. (2022).

The microstructural modifications induced by ethanol treatment align with findings in citrus pomace (Kim et al. 2024), where enzymatic and ethanol pre‐treatments significantly improved drying kinetics. In that study, the combined use of enzymes (Pectinase, Cellulase, Viscozyme) and ethanol treatment enhanced moisture removal and reduced drying time by more than 4‐fold. Although direct comparisons for apple pomace are unavailable, these results suggest that similar pre‐treatment strategies can modify plant residue structures to enhance drying efficiency.

Third, for ethanol treatment conditions, the 3 times ethanol ratio for 12 h was identified as the optimal condition. This was determined by considering both economic and efficiency factors, as extended durations or increased ethanol ratios did not yield significant improvements in drying efficiency.

Enzymatic degradation of fiber structures has also been reported in citrus by‐products, where Pectinase and Cellulase treatments enhanced moisture removal and facilitated the extraction of bioactive compounds (Hu, Kang, et al. 2021; Kim et al. 2024). This supports the effectiveness of enzyme‐assisted drying pre‐treatments in valorizing agricultural by‐products.

Fourth, in the optimization of enzyme treatment conditions, the combination of Pectinase and Cellulase was selected as the optimal enzyme treatment. This combination effectively broke down the dietary fibers in AP, thereby enhancing moisture removal. The T7_PC treatment demonstrated higher drying efficiency compared to the T5_PC treatment. The enzymatic treatment combining Pectinase and Cellulase was found to effectively degrade dietary fibers, thereby accelerating moisture removal. The role of Pectinase and other enzymes in fiber degradation and moisture removal from apple pomace has also been demonstrated in studies by Cano‐Lamadrid and Artes‐Hernandez (2021) and Martau et al. (2021).

Lastly, the optimal drying conditions were determined to be 60°C for 2 h for industrial‐scale processes and 50°C for 2 h for small‐scale applications, with both conditions ensuring effective moisture removal and high efficiency. Compared to the control, which required over 6 h at 50°C and approximately 6 h at 60°C to reach similar moisture levels, the optimized pre‐treatments reduced the drying time by over 50% at 60°C and more than 60% at 50°C while maintaining drying efficiency. This reduction in drying time significantly lowers energy consumption, enhances process efficiency, and reduces operational costs, making the approach more viable for large‐scale applications. Furthermore, shorter drying times contribute to minimizing the environmental footprint by decreasing overall energy demand and greenhouse gas emissions. These improvements highlight the potential of enzymatic and ethanol pre‐treatments as sustainable solutions for the apple processing industry.

These results imply that the pre‐treatments not only enhance drying efficiency but also contribute to significant energy savings. Previous studies have shown that drying time is a critical factor in energy consumption, where shorter drying durations directly correlate with lower electricity and thermal energy demands (Kim et al. 2024). In citrus pomace, enzymatic and ethanol pre‐treatments have been demonstrated to lower energy consumption by improving drying kinetics (Kim et al. 2024). In this study, optimized pre‐treatment conditions reduced drying time by over 50% at 60°C and more than 60% at 50°C compared to the control, which required over 6 h to reach similar moisture levels. While direct energy consumption measurements were not conducted, these drying time reductions strongly indicate improved energy efficiency. Based on previous findings, a proportional reduction in energy demand can be inferred, suggesting that pre‐treated samples require significantly less thermal input per unit of dried material.

**FIGURE 7:**
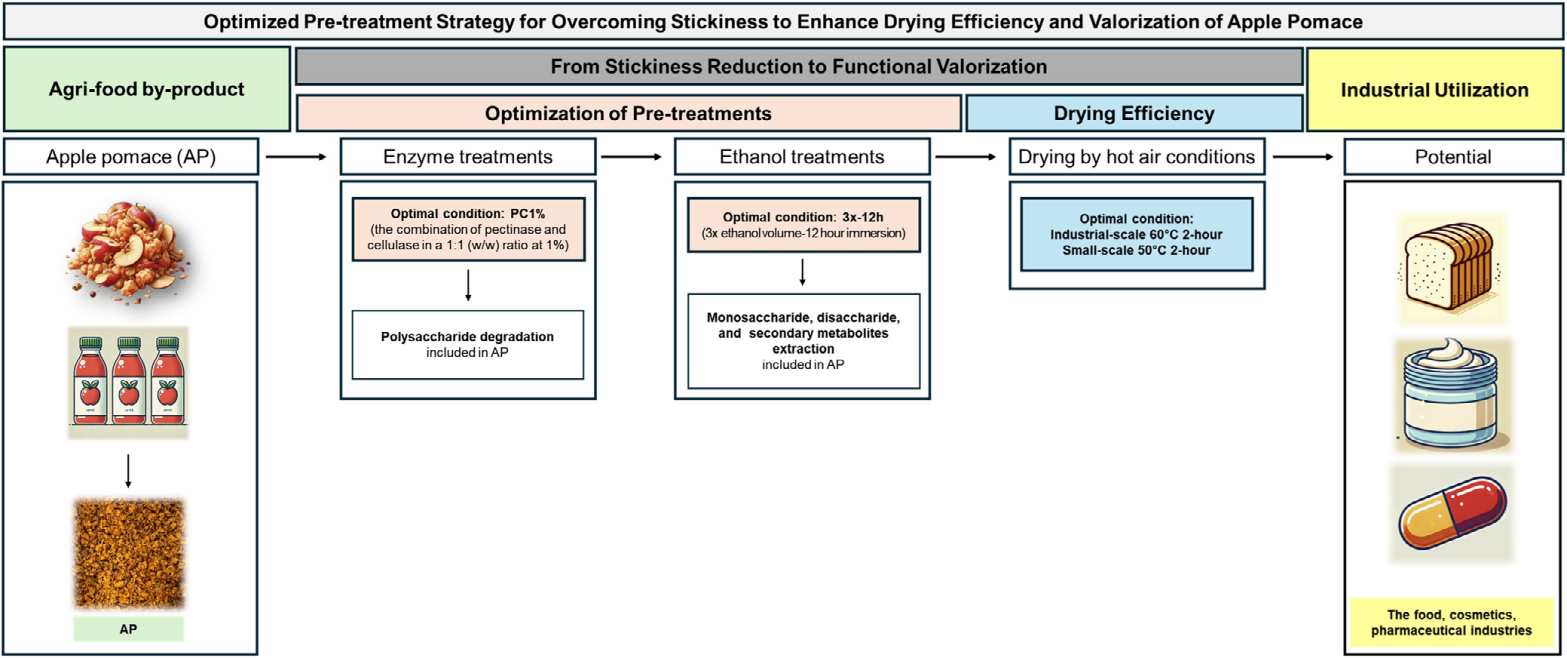
Schematic overview of stickiness mitigation and drying optimization in apple pomace valorization.

As illustrated in the Schematic overview (Figure 7), the optimized pre‐treatment and drying conditions significantly enhance the usability of apple pomace (AP), making it a valuable resource in the food, cosmetics, and pharmaceutical industries (Arraibi et al. 2021; Kotova et al. 2024; Waldbauer et al. 2017). AP, rich in dietary fiber, polyphenols, and other bioactive compounds, has diverse applications beyond food and biofuel production. Its antioxidant properties have been explored in skincare formulations and pharmaceutical applications, where AP extracts contribute to functional ingredients in nutraceuticals (Arraibi et al. 2021; Kotova et al. 2024; Manrich 2024).

While no studies have specifically optimized enzymatic and ethanol pre‐treatments for apple pomace drying, our findings help fill this research gap.

### 4.1 Sensorynutritional Quality and Future Directions

Although the primary focus of this study was to enhance drying efficiency by overcoming stickiness, recent studies have shown that enzyme‐ and ethanol‐assisted pre‐treatments can also influence the sensory and nutritional quality of dried products.

Kim et al. (2024) reported that citrus pomace subjected to a combined Viscozyme–Cellulase enzymatic treatment at 1% followed by ethanol immersion (4× *w*/*w*, 4°C, 24 h) exhibited a more than a fourfold improvement in drying efficiency (measured by removed moisture content) compared with the untreated control. Additionally, when the optimally dried sample was extracted using 40% ethanol and ultrasound at 80°C for 15 min, the combined content of narirutin and hesperidin was approximately 2.4 times higher than that obtained using conventional 70% ethanol extraction.

For apple products, Lin et al. (2022) reported that steam blanching shortened hot air drying time by ≈11%, whereas immersion in 75%–95% ethanol for 10–25 min cut drying time by ≈20%–47%. Both pretreatments enhanced total phenolic retention and limited color changes (lower ΔE) relative to untreated controls.

In contrast, Tepe (2024) reported that ethanol pretreatment of apple slices reduced drying time by up to 47% but also led to a 40%–45% loss in total phenolic content and a 17%–42% decline in antioxidant activity, indicating a clear trade‐off between efficiency and nutritional quality.

Additional studies involving ethanol‐based pre‐treatments have similarly reported improved drying kinetics but variable outcomes in phenolic compound stability and color preservation, depending on fruit type and processing conditions.

Taken together, these findings suggest that the optimized T7_PC_2 protocol has the potential to enhance drying efficiency while maintaining key quality attributes within functionally meaningful thresholds, although further validation is required.

However, to substantiate this hypothesis, future research should incorporate direct post‐drying analyses of key quality parameters, including total phenolic and flavonoid contents, HPLC‐based quantification of bioactive compounds (e.g., quercetin, phloridzin), antioxidant capacity (e.g., DPPH or ABTS assays), CIELAB color difference (ΔE), and texture profile characteristics such as hardness and cohesiveness.

In addition, pilot‐scale validation and industrial‐scale trials will be necessary to assess the technical feasibility and economic viability of implementing this process at a commercial level. Life cycle assessments (LCA) and cost–benefit analyses should be conducted to quantitatively evaluate the environmental impacts and market potential of AP‐based products. Further studies should also address the standardization of AP‐derived materials, regulatory compliance, and consumer acceptance, which will be critical for successful market integration.

Overall, this study demonstrates that the proposed pre‐treatment conditions can contribute to improving the sustainability of the apple processing industry by enhancing drying efficiency. Moreover, the findings highlight the potential of enzyme‐ and ethanol‐based pre‐treatments as scalable and sustainable technologies for industrial applications.

## 5 Conclusion

This study successfully identified optimal pre‐treatment conditions to enhance the drying efficiency of AP. By evaluating an evaluation of various ethanol concentrations, enzyme types, and treatment durations, it was determined that a combination of Pectinase and Cellulase enzyme treatments followed by ethanol treatment at room temperature (T7_PC_2) significantly improved drying efficiency. As part of this optimized pre‐treatment combination, a 3× ethanol treatment for 12 h was applied. Subsequently, the optimal drying conditions were determined to be 60°C for 2 h for industrial‐scale applications and 50°C for 2 h for small‐scale settings, which are considered effective for achieving high drying efficiency within a short processing time.

These findings suggest that the proposed pre‐treatment and drying strategies offer an effective hurdle approach to overcoming stickiness‐related challenges and improving the drying performance of apple pomace, a major agri‐food by‐product. By enhancing product stability and storability, these techniques support the sustainable preservation and valorization of food processing residues. Moreover, the developed methods hold potential for adaptation to other by‐products with similar drying limitations.

In addition, previous studies suggest that enzyme‐ and ethanol‐assisted pre‐treatments may also contribute to the retention of sensory and nutritional quality in dried products. Although the present study primarily focused on drying kinetics, further validation of key quality attributes—such as bioactive compound retention, color stability, and texture—is warranted to confirm the broader applicability of the proposed approach in food and functional ingredient systems.

## Author Contributions

**Hyo Jun Won:** data curation (equal), formal analysis (equal), investigation (equal), visualization (equal), writing – review and editing (lead). **Ae‐Jin Choi:** conceptualization (lead), funding acquisition (lead), methodology (equal), supervision (lead).

## Conflicts of Interest

The authors declare no conflicts of interest.

## Data Availability

The original contributions presented in the study are included in the article, and further inquiries can be directed to the corresponding author.
